# State of deworming coverage and equity in low-income and middle-income countries using household health surveys: a spatiotemporal cross-sectional study

**DOI:** 10.1016/S2214-109X(19)30413-9

**Published:** 2019-09-23

**Authors:** Nathan C Lo, Sam Heft-Neal, Jean T Coulibaly, Leslie Leonard, Eran Bendavid, David G Addiss

**Affiliations:** a Department of Medicine, University of California, San Francisco, San Francisco, CA, USA; b Center on Food Security and the Environment, Stanford University, Stanford, CA, USA; c Primary Care and Population Health, Stanford University, Stanford, CA, USA; d Center for Health Policy and the Center for Primary Care and Outcomes Research, Stanford University, Stanford, CA, USA; e Department of Epidemiology and Public Health, Swiss Tropical and Public Health Institute, Basel, Switzerland; f University of Basel, Basel, Switzerland; g Unité de Formation et de Recherche Biosciences, Université Félix Houphouët-Boigny, Abidjan, Côte d'Ivoire; h Centre Suisse de Recherches Scientifiques en Côte d'Ivoire, Abidjan, Côte d'Ivoire; i Focus Area for Compassion and Ethics, Task Force for Global Health, Decatur, GA, USA

## Abstract

**Background:**

Mass deworming against soil-transmitted helminthiasis, which affects 1 billion of the poorest people globally, is one of the largest public health programmes for neglected tropical diseases, and is intended to be equitable. However, the extent to which treatment programmes for deworming achieve equitable coverage across wealth class and sex is unclear and the public health metric of national deworming coverage does not include representation of equity. This study aims to measure both coverage and equity in global, national, and subnational deworming to guide future programmatic evaluation, investment, and metric design.

**Methods:**

We used nationally representative, geospatial, household data from Demographic and Health Surveys that measured mother-reported deworming in children of preschool age (12–59 months). Deworming was defined as children having received drugs for intestinal parasites in the previous 6 months before the survey. We estimated deworming coverage disaggregated by geography, wealth quintile, and sex, and computed an equity index. We examined trends in coverage and equity index across countries, within countries, and over time. We used a regression model to compute the household correlates of deworming and ecological correlates of equitable deworming.

**Findings:**

Our study included 820 883 children living in 50 countries from Africa, the Americas, Asia, and Europe that are endemic for soil-transmitted helminthiasis using 77 Demographic and Health Surveys from December, 2003, to October, 2017. In these countries, the mean deworming coverage in preschool children was estimated at 33·0% (95% CI 32·9–33·1). The subnational coverage ranged from 0·5% to 87·5%, and within-country variation was greater than between-country variation. Of the 31 countries reporting that they reached the WHO goal of more than 75% national coverage, 30 had inequity in deworming, with treatment concentrated in wealthier populations. We did not detect systematic differences in deworming equity by sex.

**Interpretation:**

Substantial inequities in mass deworming programmes are common as wealthier populations have consistently higher coverage than that of the poor, including in countries reporting to have reached the WHO goal of more than 75% national coverage. These inequities seem to be geographically heterogeneous, modestly improving over time, with no evidence of sex differences in inequity. Future reporting of deworming coverage should consider disaggregation by geography, wealth, and sex with incorporation of an equity index to complement the conventional public health metric of national deworming coverage.

**Funding:**

Bill & Melinda Gates Foundation, Stanford University Medical Scientist Training Program.

## Introduction

Soil-transmitted helminthiasis is the most prevalent neglected tropical disease affecting up to 1 billion people.^1^, ^2^, ^3^ Soil-transmitted helminthiasis affects the most disadvantaged people, helps to drive the cycle of poverty, and contributes to health inequities.^4^, ^5^ Soil-transmitted helminthiasis is defined as infection with one or more helminths from *Ascaris lumbricoides* (roundworm), *Ancylostoma duodenale* and *Necator americanus* (hookworm), and *Trichuris trichiura* (whipworm), and is associated with a range of sequelae from present or past infection, including chronic abdominal pain, anaemia, micronutrient deficiency, and stunting.^6^, ^7^, ^8^, ^9^ The global public health strategy against soil-transmitted helminthiasis is WHO-recommended deworming, often through preventive chemotherapy or mass drug administration, which is the widespread empirical treatment of at-risk populations at routine intervals. For soil-transmitted helminthiasis, deworming programmes focus on school-age (ages 5–14 years) and preschool age (ages 1–4 years) children.^10^, ^11^ Deworming programmes treat almost 600 million children annually, which makes them one of the largest public health programmes in the world.^10^, ^12^

Although soil-transmitted helminthiasis affects the most economically and socially disadvantaged, the degree to which deworming treatment programmes reach people most in need is unclear. Equitable deworming would include all individuals at risk with coverage that is generally proportional to the level of risk of soil-transmitted helminthiasis.^2^, ^13^, ^14^ Deworming is often considered a so-called pro-poor public health effort because the risk of soil-transmitted helminthiasis is likely to be higher in populations that are poorer, have lower educational status, and live in settings with inadequate sanitation.^2^, ^11^, ^13^, ^14^, ^15^, ^16^ This consideration would suggest that deworming, if equitable, would then trend towards having higher coverage in poorer populations.^2^, ^11^, ^13^, ^14^, ^15^, ^16^ The prevalence of soil-transmitted helminthiasis would likely be equal by sex given limited biological plausibility for differential risk of infection, therefore equitable deworming would be expected to have equal coverage by sex.^14^, ^15^

**Research in context**

**Evidence before this study**

We searched PubMed for relevant articles published in English from database conception to April 11, 2019, using the search terms “soil-transmitted helminth” or “deworm” or “intestinal” together with “equity” restricted to the title and abstract fields. This search identified 11 articles. None of these studies assessed the equity of deworming programmes on a subnational level or stratified by wealth and sex. We did identify an article constructing a country level, multiple neglected tropical disease index, which was computed using national coverage of multiple programmes of these diseases. We also found a multicountry study that found general equity by sex of the coverage of these programmes.

**Added value of this study**

This study provides a multicountry analysis on the state and trends in coverage and equity of deworming programmes using subnational, maternal-reported data from 820 883 preschool age children in 50 countries, which could be incorporated into routine evaluation of deworming programmes. We find that although deworming is often considered an equitable public health intervention meaning poorer populations have higher coverage, deworming coverage and equity is highly variable and medicines are generally not reaching the poor equitably (ie, wealthier populations are more likely to be treated). This variability is despite often high national coverage reported to WHO. Over time, deworming coverage by wealth seems to be modestly becoming more equal with reductions in within country variation in equity index. We found no evidence of sex-related differences in deworming equity. This study goes beyond previous work to provide a rigorous comprehensive evaluation of the coverage and equity of deworming programmes at a subnational level with disaggregation by wealth and sex using person-level household data and provides actionable information that can strengthen future measurement and evaluation of national deworming programmes.

**Implications of all the available evidence**

Deworming programmes are one of the largest public health programmes globally, and the public health metric used to track progress is national deworming coverage, which might preclude sensitive measurement of inequity. Our study findings support the opportunity for reporting of deworming coverage on a subnational level that is disaggregated by wealth and sex with opportunity to incorporate an equity index to complement routinely reported national deworming coverage. The measurement of equity in deworming programmes can be readily addressed through use of secondary datasets such as the Demographic and Health Surveys. To fully realise the potential of programmatic equity measures, the collaborative support of WHO, Ministries of Health, and non-governmental organisations will be necessary along with consideration of other public health needs in the country. Formal inclusion of subnational coverage data and an equity index will likely improve the ability of deworming programmes to address the disease burden of soil-transmitted helminthiasis affecting 1 billion of the world's poorest people.

Historically, national deworming coverage reported to WHO has been used to track progress of country programmes as the conventional metric, which follows the WHO public health goal of achieving 75% national coverage or greater.^5^ However, national deworming coverage does not necessarily reflect equity by economic status, sex, or geography. Specifically, national deworming coverage might be subject to a so-called tyranny of averages: the average coverage might appear higher despite low coverage in large geographical regions,^17^ economically disadvantaged populations, or other key subgroups. As a result, overall understanding of correlates of deworming that might be related to equity is not clear.

The goal of equity lies at the foundation of global health, although the emphasis on measuring and tracking equity in public health programmes has only increased with the Sustainable Development Goals.^18^ Literature exists, particularly for child mortality, on metrics to estimate equity for various public health programmes using summary statistics (eg, ratios) and indices (eg, concentration index).^19^, ^20^ Yet, application of these metrics to routine measurement, tracking progress over time, or setting public health targets remains limited. However, in 2016, WHO provided guidance on equity metrics, recommending disaggregating programmatic data by geography, wealth, and sex.^20^

Mass deworming has been promoted as an example of an equitable intervention in global health, yet the extent to which this assumption is true remains unclear given limited monitoring of equity with disaggregation of data or incorporation of equity measures. The aim of this study was to estimate the global status and trends in national and subnational deworming coverage and equity by wealth and sex to test the degree to which deworming for soil-transmitted helminthiasis is equitable.

## Methods

### Data and study measures

We used the Demographic and Health Surveys to examine global, national, and subnational coverage and equity in deworming, and the associated correlates of deworming. Demographic and Health Survey data are nationally representative cross-sectional surveys on demographic, health, and health system indicators that are done in over 90 low-income and middle-income countries approximately every 5 years.^21^ The Demographic and Health Surveys are funded by the US Agency for International Development, and independently implemented by ICF International to ensure consistency in data quality over time. We analysed all available Demographic and Health Surveys that included data for deworming in preschool-age children (1–4 years; 12–59 months). We excluded data at the national and subnational level in which deworming was not recommended because of low soil-transmitted helminthiasis prevalence or being non-endemic; subnational exclusion was on the basis of the geographical unit (ie, Demographic and Health Surveys region; administrative level one; appendix pp 3, 4). Estimates of drug coverage and equity were based on the most recent Demographic and Health Surveys for each country. For analyses related to temporal trends or correlates of deworming, we included all surveys when multiple Demographic and Health Surveys were available for a country. This study relied on previously collected microdata that were not individually identifiable and did not require human research approval.

The key outcome variable was deworming based on the mother's report of whether her children had received drugs for intestinal parasites in the past 6 months before the survey. When multiple children lived in the household, the mother answered separately for each child. We estimated coverage disaggregated by wealth quintile (a composite index of household assets, standardised to each survey, and computed by Demographic and Health Surveys), sex, and geography.^22^ We used Demographic and Health Survey data for prespecified variables based on a statistical plan to measure the correlates of deworming, which included: child's age and sex, mother's age (<30 years or older), mother's education (defined as no education, some primary school, or completion of primary school or higher education), wealth quintile, urban or rural residence (based on country definition, based on population or infrastructure definitions), access to improved drinking water source and to improved sanitation facility or toilets (two separate binary designations based on definitions used by the UNICEF/WHO Joint Monitoring Program^23^), and receipt of the third dose of the diphtheria, tetanus, and pertussis vaccine (DTP3) and vitamin A as generic markers of health-care access.^7^, ^24^ We excluded observations with missing data for covariates, which was an estimated 12% of the sample size (appendix pp 5, 12).

### Statistical analysis of coverage and equity

We computed deworming coverage and equity at a subnational level across and within countries and over time. We computed a measure of equity in deworming by wealth using an equity index, which was estimated using a prespecified concentration index. The concentration index is a scalar measure of equality when considering a binary outcome (eg, deworming) over a range of another socioeconomic variable (eg, composite wealth score).^25^ The generalised calculation involves the summation of binary deworming weighted by wealth quintile (normalised to zero) over the population with adjustment for overall deworming coverage and population size.^25^ We used the Erreygers correction, which provided technical advantages over other concentration indices, including satisfying a mirror condition (ie, inverse coding of the binary deworming variable would not affect the index).^25^ For interpretation, an equity index of 0 indicated that deworming was generally equally distributed across all five wealth classes. A negative equity index (bounded to a minimum of −1) indicated that deworming was concentrated in children from wealthier families; the closer to −1, the greater the concentration of deworming in children from wealthier families. A positive equity index (bounded to a maximum of 1) indicated that deworming was concentrated in children from poorer families; the closer to 1, the greater the concentration of deworming in children from poorer families. We estimated the proportion of variance in equity index, explained by within country compared with between country variation based on a previous approach with bootstrapped confidence intervals,^26^ to understand whether inequity was driven more by national policy or within country differences (appendix p 5). Methodological details on further analyses to investigate equity trends are available in the appendix pp 5, 6.

### Statistical analysis of correlates of deworming and equity

We computed the household-level correlates of deworming using all available survey data, including multiple surveys for a given country. The analysis used a multivariable logistic regression model with a dependent variable of mother-reported deworming to compute the correlates of deworming.^27^ The analysis included a prespecified set of correlates based on previous literature (appendix p 10).^7^, ^24^ We included country fixed effects, which controlled for all time-invariant differences between countries (eg, baseline country differences in economic status or soil-transmitted helminthiasis disease burden), although tested subnational region level fixed effects in sensitivity analysis. We estimated the marginal effects for correlates of deworming among children for ease of interpretation, and used robust standard errors clustered by country and survey.^28^ In the sensitivity analysis, we repeated the analysis with larger sample size with alternative variable selection (appendix p 12).

We computed the ecological correlates of equity in deworming using all available survey data averaged at a subnational level. We used an ordinary least squares regression model with a dependent variable of subnational equity index, and independent variables of region-level averages of the prespecified set of correlates as described above. We estimated the absolute change in equity index associated with a unit value for each covariate at an ecological region level. For multivariable analyses, we repeated the analysis using countries in sub-Saharan Africa alone given potential differences with deworming programmes. All analyses used R software (version 3.2.3, R Foundation for Statistical Computing; Vienna, Austria).

### Role of the funding source

The corresponding author had full access to all the data in the study and takes responsibility for the integrity of the data and the accuracy of the data analysis. The funding organisations had no role in the design and conduct of the study; collection, management, analysis, and interpretation of the data; and preparation, review, or approval of the manuscript; or the decision to submit the manuscript for publication.

## Results

The study included household survey data on 820 883 preschool age children over the period of December, 2003, to October, 2017; the most recent surveys for each country included data for 628 489 children (table 1). We included 77 Demographic and Health Surveys from 50 countries, including from Africa (n=35), the Americas (n=5), South East Asia, Eastern Mediterranean, and Western Pacific (n=9), and Europe (n=1; panel). The characteristics of our study population are shown in table 1.

**Table 1:** Characteristics of the study population of preschool children

		**Dewormed (n=227 676)**	**Not dewormed (n=400 813)**
Age (years)		2·8	2·3
Female sex		47·9%	48·6%
Male sex		52·1%	51·4%
Wealth quintile*		3·3	3·0
Children with mother's age of more than 30 years		60·0%	63·2%
Rural		66·9%	71·8%
Mother's education			
	No education	24·9%	34·1%
	Some primary school education	22·4%	22·2%
	Completion of primary school or higher education	52·7%	43·7%
Improved drinking water source		73·1%	70·1%
Improved sanitation facility or toilet		55·2%	48·3%
Receipt of 3rd dose of DPT vaccine (% received)		80·1%	61·2%
Receipt of vitamin A in past 6 months		81·1%	45·7%

Data are n or %. DTP=diphtheria, tetanus, and pertussis.

* Wealth quintiles are ordinal variables: 1, 2, 3, 4, 5.

Panel **Study countries and data from Demographic and Health Surveys by country classification based on WHO regional offices**

**Africa region**

Benin (2012); Burkina Faso (2010); Burundi (2010, 2016); Cameroon (2011); Chad (2014); Comoros (2012); Côte d'Ivoire (2012); Democratic Republic of the Congo (2007, 2013); Ethiopia (2010, 2016); Gabon (2012); Gambia (2013); Ghana (2008, 2014); Guinea (2012); Kenya (2008, 2014); Lesotho (2009, 2014); Liberia (2007, 2013); Madagascar (2008); Malawi (2010, 2015); Mali (2012); Mozambique (2011); Namibia (2006, 2013); Niger (2012); Nigeria (2008, 2013); Republic of the Congo (2011); Rwanda (2008, 2010, 2014); São Tomé and Príncipe (2008); Senegal (2010); Sierra Leone (2013); South Africa (2016); eSwatini, previously known as Swaziland (2006); Tanzania (2010, 2015); Togo (2013); Uganda (2006, 2011, 2016); Zambia (2007, 2013); Zimbabwe (2010, 2015)

**Region of the Americas**

Dominican Republic (2007, 2013); Guyana (2009); Haiti (2006, 2012, 2016); Honduras (2011); Peru (2004)

**South-East Asia region**

Bangladesh (2011, 2014); India (2015); Myanmar (2015); Nepal (2006, 2011, 2016); Timor-Leste (2009, 2016)

**European region**

Azerbaijan (2006)

**Eastern Mediterranean region**

Pakistan (2012); Yemen (2013)

**Western Pacific region**

Cambodia (2005, 2010, 2014); Philippines (2008, 2017)

**Figure 1:**
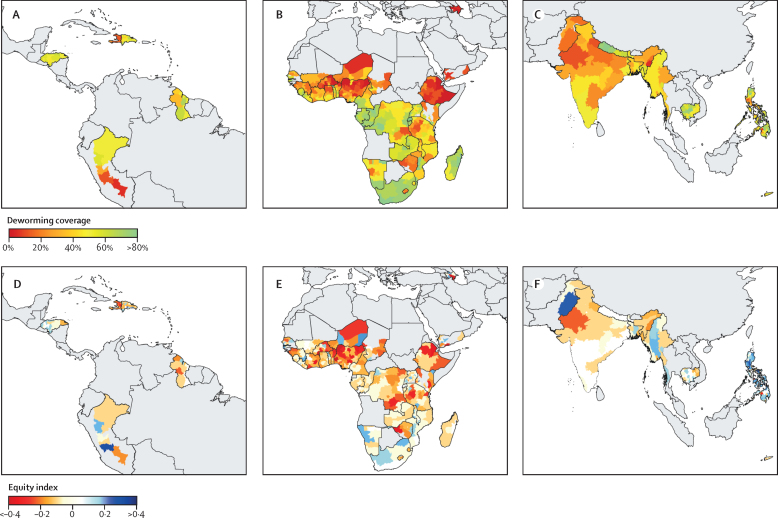
Maternal-reported deworming coverage and equity in pre-school children in low- and middle-income countries We estimated subnational deworming coverage (A, B, C) and the equity index (D, E, F) for all study countries. The map was geographically divided into the Americas (A, D), Africa (B, E), and South East Asia, Eastern Mediterranean, and Western Pacific (C, F) for data visualisation purposes. For the interpretation of the equity index, a negative index indicated that deworming was concentrated in wealthier households on average, whereas a positive index indicated that deworming was concentrated in poorer households. Greyed areas of the map are where data are not available or soil-transmitted helminths are not endemic.

Across all countries and years, 33·0% (95% CI 32·9–33·1) of mothers reported that their child received deworming in the past 6 months. Deworming coverage varied substantially across countries, with an even higher degree of within-country variation (figure 1). Deworming coverage in areas that are endemic for soil-transmitted helminthiasis varied by country, from 5·1% (Azerbaijan) to 73·6% (Rwanda) and varied further at the subnational (regional) level, which ranged from 0·5% (Zamfara, Nigeria) to 87·5% (Kavango, Namibia). The estimated proportion of variation in deworming coverage explained by within-country factors (eg, state policies, variation in wealth and health-care access within a country, etc) was 90·8% (95% CI 90·6–90·9), whereas the remaining 9·2% (9·0–9·4) was attributable to between-country factors (eg, national policies and programmes). The national deworming coverage reported to WHO was greater than the coverage estimated by Demographic and Health Survey data in 40 of 45 cases, with a mean difference of 36% (range −35 to 75).

**Figure 2:**
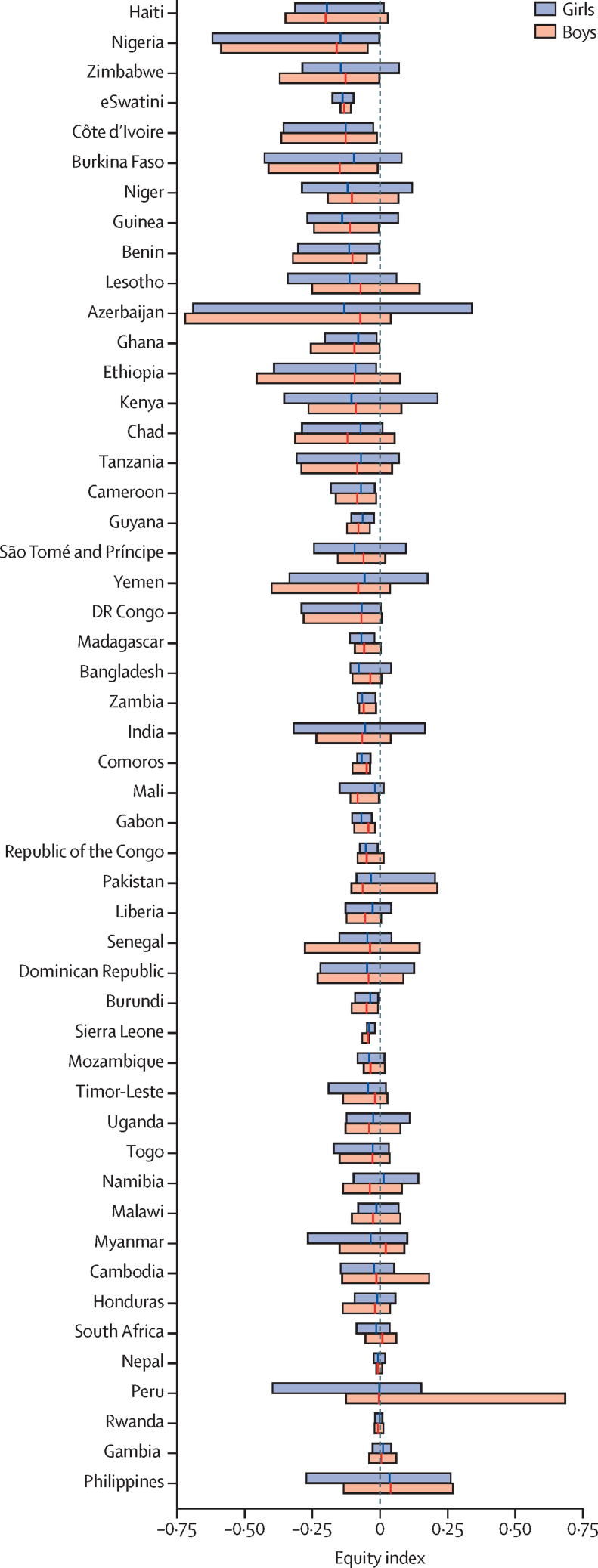
Geographical variation in maternal-reported deworming equity by country and sex We estimated an equity index for deworming at a subnational level for each study country, which provided data on within country variation of deworming equity. The analysis was conducted independently by sex for comparison of equity by sex for each study country. The width of each box provides the 2·5th and 97·5th percentile of the equity index computed for each country. For interpretation, the width of each bar corresponds with the magnitude of within country variation in equity; in which a negative index indicated deworming was concentrated in wealthier households, whereas a positive index indicated deworming was concentrated in poorer households. The vertical line in each bar represents the mean equity index.

In our study population, deworming of preschool children increased linearly with household wealth, ranging from deworming coverage of 27·5% (95% CI 27·3–27·7) for children in the lowest wealth quintile to 38·3% (38·1–38·5) in the highest quintile. Of the 50 countries, 14 had a negative equity index in every endemic region of the country (eg, in Nigeria, we found a 28% absolute difference in deworming coverage between children in the lowest and highest wealth quintile). At a subnational level, some countries had substantial within-country variation in the equity index, including Azerbaijan, Burkina Faso, India, Nigeria, Peru, and Philippines. Of the 31 study countries reported to WHO to have achieved a national deworming coverage greater than the global public health goal of 75% (appendix pp 9), we found that 30 had a negative mean equity index. We calculated equity index independently by sex, and notably did not find substantial systematic differences between boys and girls in deworming equity index across study countries (figure 2).

**Figure 3:**
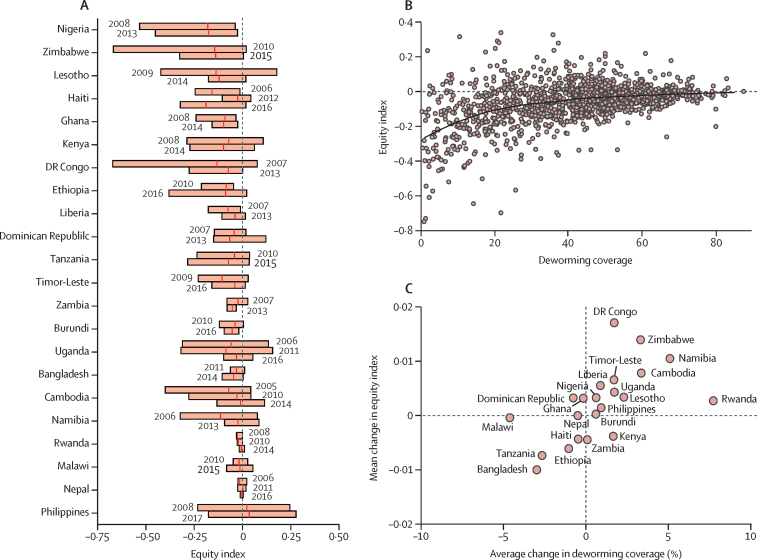
Trends in maternal-reported deworming equity over time and by programmatic coverage (A) We estimated an equity index for deworming in countries with surveys at different time periods, which provided data for country trends for geographical variation in deworming equity. The width of each box provides the 2·5th and 97·5th percentile of the equity index computed for each country. The bars are grouped by country, with more recent surveys shown on the lower bars. For interpretation, deworming equity was considered to improve for a country with a reduced width of the bar (reduced within country variation in equity) or a mean index (vertical line in bars) closer to zero (equal across wealth classes). (B) We fitted the relationship for subnational mean deworming coverage and equity index for all study countries. (C) We estimated the discrete annualised change in mean deworming coverage and the corresponding annualised change in equity index for study countries with surveys at different time periods.

For countries with multiple Demographic and Health Surveys, we found modest improvements in deworming coverage and equity across wealth classes over time. We found an average increase in these countries in national deworming coverage by 0·7% annually. For temporal trends in the equity index, we estimated that eight countries had notable improvements in deworming equity, ten countries had no changes, and four countries became less equitable (appendix p 8 for definitions). We found a qualitative trend at the subnational level that regions with higher coverage had a less negative equity index (figure 3). When estimated subnational deworming coverage approached 70% using study coverage estimates, the mean equity index approached zero (ie, suggesting equal distribution of deworming across wealth classes). Similarly, when we estimated how subnational changes in mean deworming coverage were associated with corresponding changes in equity index, we found that higher coverage was related to a modestly more positive equity index (figure 3).

In a multivariable analysis, we found that medicine for deworming during the previous 6 months was most strongly associated with vitamin A supplementation and DPT3 vaccine, child's age, and maternal education (table 2). We found weaker associations between deworming and greater family wealth and improved toilets. There was a modest relationship between deworming and sex (adjusted marginal effects −0·5, 95% CI −0·7 to −0·2), which corresponded with a 0·5% lower coverage in girls (table 2). In an ecological multivariable analysis, we found a positive equity index was most strongly associated with high coverage of DTP3 vaccine and vitamin A supplementation, average child age, maternal education, and wealthier populations at a subnational level (appendix p 11). The estimated explanatory value of sex on the variation in deworming equity was less than 1%. When examining sub-Saharan African countries alone, we found these relationships to be generally consistent with some differences for ecological correlates of equity (appendix pp 13, 14). The results were generally robust to alternative variable selection to reduce missing data and alternative statistical specifications (appendix pp 12, 15).

**Table 2:** Correlates of maternal-reported deworming in preschool age children

	**Adjusted marginal effects**	**95% CI**
Age (year)	6·3	4·0 to 8·6*
Female sex	−0·5	−0·7 to −0·2*
Wealth quintile	1·2	0·6 to 1·7*
Children with mother's age of more than 30 years	−0·8	−1·3 to −0·4*
Rural residence	0·6	−0·2 to 1·5
Mother's education (tertile)†	3·3	2·5 to 4·0*
Improved drinking water source	0·6	−0·4 to 1·6
Improved sanitation facility or toilet	1·4	0·5 to 2·2*
3rd dose of DTP vaccine	10·0	7·5 to 12·6*
Vitamin A	33·1	30·6 to 35·6*

Marginal effects refer to absolute change in mean deworming coverage associated with one unit change in variable. Robust standard errors clustered by country and survey. DTP=diphtheria, tetanus, and pertussis.

* Indicates a relationship with a p<0·05.

† Is this the marginal effect obtained by 1 unit change in the mother's education.

## Discussion

In this empirical study, we used cross-sectional household survey data on maternal-reported deworming in 820 883 preschool age children in 50 countries to estimate the global status and trends in coverage and equity for deworming programmes. Although deworming is intended to further the goal of health equity, in most countries, deworming coverage is highly geographically variable and deworming coverage was consistently higher in wealthier populations. We do not find systematic evidence of sex-related differences in the equity index. Of the 31 study countries that reported a greater than 75% national coverage to WHO, 30 had a negative equity index for deworming (greater coverage among wealthier populations). Current coverage metrics that report mean national coverage would not detect this inequity. Over time, deworming coverage by wealth seems to be modestly improving to become equal across wealth classes, along with associated reductions of within-country variation in equity. The strongest correlates of deworming, as well as a positive equity index, were vitamin A supplementation and DTP3 vaccine, which are markers of health-care access that might attenuate the effects of poverty. The findings from our global empirical analysis of cross-sectional household surveys support the need for measurement of equity in national deworming programmes and shows the potential to incorporate secondary data sources into the measurement framework.

The goal of equity lies at the foundation of global health, despite the challenge of achieving it.^29^ Measurement of equity has become increasingly considered in the evaluation of global health programmes. Goal 10 of the Sustainable Development Goals set in 2015 is to reduce inequities, and WHO now includes equity as a core consideration in their evaluation of evidence and guidelines.^18^ Analyses of global health programmes have addressed equity, for example in the case of DTP3 vaccination where data showed persistent, but decreasing, inequity.^30^ An equity index for multiple neglected tropical diseases of national programmes has also been developed.^31^ However, most of these analyses have examined trends at the national rather than subnational level. They have not disaggregated by wealth and sex, which limits interpretation and development of an equity metric that can be incorporated into programmatic use.

Deworming is often stated to be so-called pro-poor given that soil-transmitted helminthiasis and other neglected tropical diseases most commonly affect the poor.^2^, ^11^, ^13^, ^14^, ^15^, ^16^ However, an intervention such as deworming would not be pro-poor if, despite high burden among poor people, empirical evidence finds coverage is concentrated among the wealthy. The empirical evidence for this issue was explicitly tested with our analysis. The definition of equity in health interventions is complex, and in this study, we define equity as deworming coverage that is generally proportional to risk of soil-transmitted helminthiasis. In that context, the numerical range for the equity index that represents equitable deworming coverage is likely to be modestly positive. This value would reflect the modestly (but not extremely) higher prevalence and transmission of soil-transmitted helminthiasis in poorer population. We did not find strong evidence that deworming is consistently pro-poor; the deworming equity index was negative in most settings, meaning that deworming is more concentrated in wealthier populations. In most countries, national deworming coverage estimated by maternal-reported Demographic and Health Survey data was also substantially lower than coverage data reported to WHO, which might reflect both limitations in obtaining self-reported deworming status or robustness in national reporting of deworming coverage. No clear differences were noted in deworming equity by sex. However, this finding does not exclude the possibility of sex inequity in many settings and supports the need for ongoing monitoring of sex equity.^32^ As deworming coverage increased, the equity index trended towards higher relative coverage in poorer populations, suggesting some progress towards achieving pro-poor coverage is feasible.

Historically, the key metric in deworming for defining public health goals and monitoring programmatic progress has been national coverage. WHO defines the public health goal for soil-transmitted helminthiasis as achieving greater than 75% national coverage of deworming in preschool and school-age children. However, overall national coverage estimates can appear high while substantial inequities persist across geography, wealth, and sex (a concept referred to as the tyranny of averages).^17^ Many countries have substantial variation in deworming equity within the country, which is not captured with national coverage alone, while other countries have little within-country variation. The large subnational variation within some countries might represent subnational differences in robustness of programme coverage and health-care access, although it might also be influenced by size of the country and number of regions that report coverage.^14^, ^31^ As the largest variations in coverage are not explained at a national level (eg, country guidelines, national policy), this finding might underscore the importance of reporting subnational coverage data. Notably, deworming coverage data is often monitored and reported on a subnational level by programme managers and Ministries of Health, although these data are not used to set current WHO public health goals. This analysis lends support to efforts to use subnational data to estimate equity of coverage across demographic groups (eg, wealth and sex) to guide public health programmes. In practice, routine reporting of programmatic coverage can be complemented by an equity index based on Demographic and Health Surveys data.

We estimated that the strongest household correlates of deworming receipt were vitamin A supplementation and DTP3 vaccine. The relationship with vitamin A is probably related to the common co-administration of deworming and vitamin A in preschool children in mass community-based campaigns, whereas the relationship with DTP3 (ie, completion of all three doses) is more a marker of access to general health services (both household and systems-level indicator) and access to health information given a mother's education level than a causal factor. The strongest ecological predictors of a positive deworming equity index shared these characteristics and were identified as subnational regions with higher coverage of DTP3 and vitamin A supplementation. The data suggest that where strong health-care services exist, such as routine health care (eg, three doses of DTP3) and public health programmes (vitamin A provision), deworming equity is likely to be greater, although the relationship can be complex.^14^, ^29^, ^31^, ^32^

The study has several limitations. The main outcome variable, deworming during the past 6 months, was proxy-reported by the mother and was subject to recall bias, which could in theory vary by maternal wealth. However, previously published data suggest that the reliability of self-reported and mother proxy-report for deworming through mass drug administration is reasonably good. These data would support the use of mother-reported deworming data from Demographic and Health Surveys, although this study^33^ was from a single site and highlighted challenge of when multiple pills were given. The proxy-reported deworming variable in this study would have included both programmatic deworming (eg, preventive chemotherapy, through child health days) and unprogrammed deworming (eg, treatment from the local pharmacy or health-care centre).^34^, ^35^ However, deworming is commonly given with vitamin A supplements during child health days; the fact that 82% of children dewormed during the previous 6 months also were reported to have received vitamin A supplements during the same timeframe suggests that most deworming occurred in the context of preventive chemotherapy. Importantly, wealthier households might be more likely to purchase or obtain deworming medication in an unprogrammed setting (eg, health clinic and pharmacy), which would bias study findings towards an inequitable distribution (ie, higher coverage in wealthier populations) when measuring equity for programmatic deworming. Furthermore, maternal reports might misclassify the type or purpose of drugs given during child health days, which could underestimate deworming (eg, child could receive albendazole for empirical treatment of lymphatic filariasis, and not recognise its dual role as a deworming medicine) or over-estimate deworming (eg, child could receive vitamin A supplementation and report this intervention as deworming given common co-administration). The nationally representative nature of Demographic and Health Surveys data and predetermined sample size limit the granularity of subnational analyses, thus the geographical regions in this study are often larger than the unit of programmatic decision making. There are alternative mathematical formulations for the equity index that can affect this estimate, therefore relative comparison of equity index for trends are more robust than use of the absolute value of the index. We excluded geographical regions (national and subnational) known to be non-endemic for soil-transmitted helminthiasis or to have sufficiently low prevalence so that preventive chemotherapy is not recommended, although up-to-date data on soil-transmitted helminthiasis prevalence and geographical coverage of national deworming programmes were not always complete (appendix pp 3,4).

In conclusion, deworming coverage and equity in children of preschool age is highly variable, which is not evident in national data reported to WHO. Specifically, in most countries, deworming does not reach the poor equally despite deworming being considered an equitable intervention. Demographic and Health Survey data can be used to inform the equity of programmatic deworming to address the global burden of soil-transmitted helminthiasis.
