# Supplementary material for: State of deworming coverage and equity in low-income and middle-income countries using household health surveys: a spatiotemporal cross-sectional study
Source: Lancet Glob Health. 2019 Sep 23;7(11):e1511–20. doi: 10.1016/S2214-109X(19)30413-9 (PMC7024997; doi:10.1016/S2214-109X(19)30413-9)
Supplement: Supplementary appendix [file mmc1.pdf]

THE LANCET
Global Health

Supplementary appendix

This appendix formed part of the original submission and has been peer reviewed. We post it as supplied by the authors.

Supplement to: C Lo NC, Heft-Neal S, Coulibaly JT, Leonard L, Bendavid E, Addiss DG. State of deworming coverage and equity in low-income and middle-income countries using household health surveys: a spatiotemporal cross-sectional study. *Lancet Glob Health* 2019; published online Sept 23. https://doi.org/10.1016/S2214-109X(19)30413-9.

Supplemental Materials

Lo NC, Heft-Neal S, Coulibaly JT, Leonard L, Bendavid E, and Addiss DG. State of deworming coverage and equity in low-income and middle-income countries using household health surveys: a spatiotemporal cross-sectional study. Lancet Global Health (2019).

Contents

Section 1: Technical appendix……………..…………….……..……..………………….Page 3

Section 2: Supplemental figures and tables…………………..…………….…………......…Page 7

Section 1: Technical appendix

In this technical appendix, we provide further description of data processing of the Demographic and Health Surveys (DHS) and statistical analysis for the study.

<u>*Selection of DHS surveys*</u>
We included all DHS surveys with data on deworming receipt in pre-school children (variable h43), as proxy-reported by the mother (see Methods). The survey question was formulated as whether the child received "drugs for intestinal parasites in the 6 months prior to the survey".

We excluded DHS surveys from countries non-endemic for soil-transmitted helminthiasis (STH) or with low STH prevalence where mass deworming would not be recommended per WHO guidelines.[1] For STH-endemic study countries, we further excluded data on a sub-national basis in regions (administrative level one) due to low STH prevalence in majority of geographic zone where deworming would not be recommended per WHO guidelines. The exclusion process was determined using the WHO preventive chemotherapy and transmission control databank, recent STH mapping data from the Expanded Special Project for Elimination of NTDs (ESPEN), and other sources, and, in some cases, correspondence with national NTD program managers or WHO NTD focal points when these data sources were insufficient.[2,3] We excluded data in STH-endemic countries on a sub-national basis using the process outlined below:

1. For all countries in sub-Saharan Africa, we used the ESPEN database when available,[3] which included results of recent neglected tropical disease (NTD) mapping at the district level. We excluded all districts that were indicated in green as "non-endemic," i.e., <1% STH prevalence.

2. For all countries not included in the ESPEN database, we used the WHO preventive chemotherapy (PCT) databank[2] and UN Population and Vital Statistics Report to estimate the proportion of pre-school age children (ages 1-4 years) eligible for deworming, defined as: $a = \frac{Pop_{PCT,preschool}}{Pop_{preschool}}$. For countries (a >0.90), we considered the entire country to be endemic; for countries (a <0.90), see step 3. The threshold of 0.90 was selected to be approximating 100% of the country within the margin of expected measurement error between datasets.

3. For countries (a <0.90), we repeated the process in Step 1 using the school-age children (ages 5-14 years) population, where $b = \frac{Pop_{PCT,schoolage}}{Pop_{schoolage}}$. As data reporting has been historically more developed for this age population and risks are comparable between the age groups, this step was included to avoid removal of data from endemic countries due to poor quality reporting. For countries (b >0.90), we considered the entire country to be endemic; for countries (b <0.90), see step 4.

4. For countries (a <0.90) and (b <0.90), we consulted available STH endemicity maps to exclude non-endemic or low prevalence geographic zones of endemic countries on a sub-national basis. We used available data from NTD Mapper, Global NTD database, GAHI, and

peer-reviewed literature. Where questions remained, we contacted other sources, including: WHO, NTD contacts in WHO regional offices, national STH program managers, Children Without Worms, and the NTD Supply Chain Forum database to make final decisions.

5. For sub-national geographic zones (DHS regions) with majority (defined as >50%) of area considered endemic, these were included in the analysis. Otherwise, these regions were excluded.

The process for inclusion and exclusion of DHS data was defined prior to the analysis being conducted. Table A1 includes DHS survey data excluded from the analysis.

Table A1: DHS surveys excluded from study

Country	Survey years	Comments	Source
Albania	2008	Not in PCT Databank	WHO PCT Databank
Armenia	2010	Low endemicity; mapping data inadequate to include specific areas	WHO
Bolivia	2008	Mapping data inadequate to include specific areas	WHO
Egypt	2014	“No PC required”	WHO PCT Databank
Kyrgyzstan	2012	Mapping data inadequate; low endemicity	WHO
Maldives	2009	”No PC required”	WHO PCT Databank
Tajikistan	2012	Low endemicity; mapping data inadequate to include specific areas	WHO

STH, soil-transmitted helminthiasis; PCT, preventive chemotherapy and transmission control

Table A2: Countries with non-endemic regions excluded from study

Country	Survey years	Comments	Source
Chad	2014	Partially endemic	ESPEN
Democratic Republic of Congo	2007, 2013	Partially endemic	ESPEN
Ethiopia	2010, 2016	Partially endemic	ESPEN
Gambia	2013	Partially endemic	ESPEN
Kenya	2008, 2014	Partially endemic	ESPEN
Madagascar	2008	Partially endemic	ESPEN
Namibia	2006, 2013	Partially endemic	ESPEN
Nigeria	2008, 2013	Partially endemic	ESPEN
Pakistan	2012-13	Partially endemic	WHO; unpublished mapping data
Peru	2004	Partially endemic	WHO; published literature
Senegal	2010	Partially endemic	ESPEN
Swaziland	2006	Partially endemic	ESPEN
Tanzania	2010, 2015	Partially endemic	ESPEN
Yemen	2013	Partially endemic	WHO; unpublished mapping data
Zimbabwe	2010, 2015	Partially endemic	ESPEN

Statistical analysis
We estimated the proportion of variance in equity index explained by within-country compared to between-country variation to understand whether equity was driven more by national policy or within country differences. To estimate the proportion of variation explained between country differences, we performed a logistic regression with deworming variable as the independent variable and country indicator variables as the dependent variables and estimated the R^2 value; the remainder of this R^2 value were attributable to within country differences as the between country differences were accounted for with the indicator variables. We used bootstrapping to generate 95% confidence intervals.

We used a local smoothed regression to qualitatively summarize the relationship between regional deworming coverage and equity index, and also examined the relationship of discrete changes in coverage and equity over time. We compared national deworming coverage estimates reported to WHO and estimated by DHS in study countries, and estimated the number of countries that reported to WHO a national deworming coverage of >75% in pre-school children.

For construction of 95% confidence intervals (95% CI) for deworming, we computed standard errors for a binomial proportion using the DHS survey weights. For the global estimate, we weighted this standard error by population size of each country.

Missing data
The DHS surveys included in the study are shown in Table 1 of the main text, and the process for survey inclusion and exclusion is described in the Methods and in the technical appendix as above. The key study variable was the proxy-reported survey question on receipt of deworming in pre-school children. We included observations marked “yes” and “no”, and excluded observations for this variable that were missing or marked as “don’t know,” “not reported,” etc. In the predictors of deworming analysis, we only included children with complete observation record for deworming receipt variable and defined predictors. In sensitivity analysis, we repeated the analysis with larger sample size by omitting variables with higher proportion of missing data, including receipt of vitamin A, DPT3, and maternal education.

Analytic code
We used R software (R Foundation for Statistical Computing; Vienna, Austria). The authors fully support the importance of data sharing and transparency in research, and analytic code are available on request to the corresponding author. All data files are available from the DHS database online.

Additional results and discussion
When considering healthcare access, we found that of the 67% (SE: 0.04%) of pre-school children who completed their DTP3 vaccine, only 38% (SE: 0.05%) of these children had received deworming in the previous 6 months; deworming coverage was estimated at 19% (SE: 0.06%) for children who did not receive their DTP3 vaccine (third dose). Similarly, of the 58% (SE: 0.04%) of pre-school children who had received vitamin A supplementation in the previous 6 months, 47% (SE: 0.06%) of these children had also been dewormed. We estimated 82% of children dewormed during the previous 6 months also were reported to have received vitamin A supplements.

These results identify the potential to integrate deworming efforts with other interventions (e.g. vitamin A) that have achieved high coverage where deworming coverage is persistently inequitable or low.

References

1. WHO. Helminth control in school-age children: a guide for managers of control programmes: Geneva: World Health Organization 2011.
2. WHO Neglected tropical diseases: PCT databank (Soil-transmitted helminthiases). http://www.who.int/neglected_diseases/preventive_chemotherapy/lf/en/ (accessed 12/24/18.
3. WHO AFRO: Expanded Special Project for Elimination of Neglected Tropical Diseases (ESPEN). 2018. http://espen.afro.who.int (accessed 12/24/18.

Section 2: Supplemental Figures and Tables

Table S1: Trends in equity by study country over time

Table S2: Study countries and national deworming coverage in pre-school children reported to WHO and estimated by DHS

Table S3: Variables in regression model on predictors of deworming receipt

Table S4: Regression model estimating the ecological correlates of deworming equity in pre-school age children

Table S5: Correlates of maternal-reported deworming in pre-school age children for subset of covariates with limited missing data

Table S6: Correlates of maternal-reported deworming in pre-school age children in sub-Saharan Africa

Table S7: Regression model estimating the ecological correlates of deworming equity in pre-school age children in Sub-Saharan Africa

Table S8: Correlates of maternal-reported deworming in pre-school age children with region level fixed effects

Table S1: Trends in equity by study country over time

Study country	Survey year	Equity trend[a]
**Cambodia**	2005, 2010, 2014	**Improved**
**DR Congo**	2007, 2013	**Improved**
**Lesotho**	2009, 2014	**Improved**
**Liberia**	2007, 2013	**Improved**
**Namibia**	2006, 2013	**Improved**
**Timor-Leste**	2009, 2016	**Improved**
**Uganda**	2006, 2011, 2016	**Improved**
**Zimbabwe**	2010, 2015	**Improved**
**Burundi**	2010, 2016	**Unchanged**
**Dominican Republic**	2007, 2013	**Unchanged**
**Ghana**	2008, 2014	**Unchanged**
**Kenya**	2008, 2014	**Unchanged**
**Malawi**	2010, 2015	**Unchanged**
**Nepal**	2006, 2011, 2016	**Unchanged**
**Nigeria**	2008, 2013	**Unchanged**
**Philippines**	2008, 2017	**Unchanged**
**Rwanda**	2008, 2010, 2014	**Unchanged**
**Tanzania**	2010, 2015	**Unchanged**
**Bangladesh**	2011, 2014	**Worsened**
**Ethiopia**	2010, 2016	**Worsened**
**Haiti**	2006, 2012, 2016	**Worsened**
**Zambia**	2007, 2013	**Worsened**

[a]Equity trend designation is based on qualitative data interpretation; changes in equity trend are observed with change in mean equity index and/or height of bar (variation in equity within country).

Table S2: Study countries and national deworming coverage in pre-school children reported to WHO and estimated by DHS

Study country	Survey year	WHO national coverage	DHS national coverage	Study country	Survey year	WHO national coverage	DHS national coverage
**Africa Region**				**Africa Region**			
Benin	2012	100**	47.2	Tanzania	2015	100	34.4
Burkina Faso	2010	-	12.2	Togo	2013	100	45.5
Burundi	2016	94.0	61.4	Uganda	2016	59.7	56.1
Cameroon	2011	100	47.7	Zambia	2013	97.5	55.7
Chad	2014	100	28.5	Zimbabwe	2015	25.6	18.9
Comoros	2012	100*	52.8				
Côte d'Ivoire	2012	100**	35.1	**Americas Region**			
DR Congo	2013	57.9	56.6	Dominican Republic	2013	33.6	48.5
Ethiopia	2016	72.3*	12.3	Guyana	2009	77.3	48.8
Gabon	2012	100	68.5	Haiti	2016	74.6	14.4
Gambia	2013	100	34.1	Honduras	2011	26.6	49.5
Ghana	2014	-	34.9	Peru	2004	-	29.8
Guinea	2012	100*	26.1				
Kenya	2014	42.0	39.8	**South-East Asia Region**			
Lesotho	2014	-	21.0	Bangladesh	2014	1.9	36.6
Liberia	2013	100	52.7	India	2015	67.6	30.7
Madagascar	2008	100	63.6	Myanmar	2015	95.5	38.7
Malawi	2015	83.1*	41.5	Nepal	2016	86.5	60.7
Mali	2012	50.0**	29.6	Timor-Leste	2016	100	44.4
Mozambique	2011	100	42.0				
Namibia	2013	89.6	44.9	**European Region**			
Niger	2012	100	25.0	Azerbaijan	2006	2.9*	5.0
Nigeria	2013	36.6	18.7				
Republic of the Congo	2011	100*	69.8	**Eastern Mediterranean Region**			
Rwanda	2014	100	73.5	Pakistan	2012	92.2*	24.9
Sao Tome and Principe	2008	24.9**	50.1	Yemen	2013	-	10.4
Senegal	2010	100	50.5				
Sierra Leone	2013	100	53.8	**Western Pacific Region**			
South Africa	2016	100	61.1	Cambodia	2014	96.2	53.7
Swaziland	2006	100	43.4	Philippines	2017	80.8	39.5

WHO national coverage data for deworming in pre-school children was obtained from the WHO preventive chemotherapy databank, which was reported as the proportion of the population requiring preventive chemotherapy for STH in the country that have been treated. We obtained WHO data corresponding to the year of the most recent DHS survey, or the most recent available data within one year of DHS survey (indicated by *) or within two years (indicated by **). When two coverage estimates were provided in the WHO databank for national coverage in a given year, we used the higher value. When data were available for both earlier and later years, an average was taken to estimate coverage in the survey year. DHS national coverage was estimated directly from survey data as described in the Methods.

Table S3: Variables in regression model on predictors of deworming receipt

Variable	Category	Variable
Child’s age	Demographic	Categorical
Child’s gender	Demographic	Binary
Wealth quintile	Socioeconomic	Continuous
Residence	Demographic	Binary (rural/urban)
Mother’s age	Demographic	Binary (<30 years)
Mother’s education	Socioeconomic	Categorical; 3-categories (None, any up to primary, beyond primary)
Improved water access	Socioeconomic	Binary
Improved toilet access	Socioeconomic	Binary
Received 3rd dose diphtheria-pertussis-tetanus vaccine	Healthcare	Binary
Vitamin A in past 6 months	Healthcare	Binary

Table S4: Regression model estimating the ecological correlates of deworming equity in pre-school age children

Variable	Coefficient	95% CI
Age (year)	**0.09**	**(0.02,0.15)**
Gender (female)	-0.1	(-0.31,0.10)
Wealth quintile	**0.01**	**(0.00,0.02)**
Mother's age (<30 years)	-0.03	(-0.11,0.05)
Rural residence	0.03	(-0.02,0.08)
Mother's education (tertile)	**0.03**	**(0.01,0.06)**
Improved drinking water	0.04	(-0.01,0.1)
Improved toilet facility	0.02	(-0.04,0.07)
Receipt of 3rd dose of DPT vaccine	**0.08**	**(0.00,0.17)**
Receipt of vitamin A supplementation	**0.15**	**(0.09,0.22)**

Bolded findings indicate a relationship with a p<0.05. The coefficient refers to absolute change in equity index associated with one unit change in the variable.

Table S5: Correlates of maternal-reported deworming in pre-school age children for subset of covariates with limited missing data

Variable	Adjusted marginal effects	95% CI
Age (year)	**7·7**	**(5·2, 10·2)**
Sex (female)	**-0·4**	**(-0·6, -0·3)**
Wealth quintile	**3·3**	**(2·3, 4·3)**
Mother's age (<30 years)	-0·3	(-1·4, 0·8)
Rural residence	1·2	(-0·1, 2·5)
Improved drinking water	**1·7**	**(0·5, 2·9)**
Improved toilet facility	1·4	(-0·4, 3·2)

Bolded findings indicate a relationship with a p<0·05. Marginal effects refer to absolute change in mean deworming coverage associated with one unit change in variable. Robust standard errors clustered by country and survey. Coefficients in Table S5 are estimated the same as in Table 3 in the main text but without mother's education, vitamin A, and 3rd dose of DTP, each of which have missing values in the data.

Table S6: Correlates of maternal-reported deworming in pre-school age children in sub-Saharan Africa

Variable	Adjusted marginal effects	95% CI
Age (year)	**7·8**	**(6·2, 9·5)**
Sex (female)	-0·5	(-0·9, 0·0)
Wealth quintile	**2·0**	**(0·9, 3·2)**
Mother's age (<30 years)	**-1·3**	**(-2·0, -0·6)**
Rural residence	0·6	(-0·9, 2·1)
Mother's education (tertile)	**4·3**	**(2·8, 5·8)**
Improved drinking water	1·3	(-0·1, 2·7)
Improved toilet facility	1·2	(-0·2, 2·6)
3rd dose of DTP vaccine	**13·3**	**(11.5, 15·0)**
Vitamin A	**35·8**	**(31·8, 39·9)**

Bolded findings indicate a relationship with a p<0·05. Marginal effects refer to absolute change in mean deworming coverage associated with one unit change in variable. Robust standard errors clustered by country and survey. See Methods for statistical specifications. DTP; diphtheria, tetanus, and pertussis

Table S7: Regression model estimating the ecological correlates of deworming equity in pre-school age children in Sub-Saharan Africa

Variable	Coefficient	95% CI
Age (year)	0.06	(-0.04,0.16)
Gender (female)	0.03	(-0.20,0.26)
Wealth quintile	**0.03**	**(0.01,0.04)**
Mother's age (<30 years)	0.06	(-0.02,0.15)
Rural residence	**0.07**	**(0.02,0.11)**
Mother's education (tertile)	0.02	(-0.02,0.06)
Improved drinking water	0.04	(-0.03,0.1)
Improved toilet facility	-0.02	(-0.09,0.05)
Receipt of 3rd dose of DPT vaccine	0.08	(-0.03,0.19)
Receipt of vitamin A supplementation	**0.16**	**(0.07,0.24)**

Bolded findings indicate a relationship with a p<0.05. The coefficient refers to absolute change in equity index associated with one unit change in the variable.

Table S8: Correlates of maternal-reported deworming in pre-school age children with region level fixed effects

Variable	Adjusted marginal effects	95% CI
Age (year)	**7.4**	**(6.2, 8.5)**
Sex (female)	-0.3	(-1.5, 0.9)
Wealth quintile	-1.5	(-3.1, 0.2)
Mother's age (<30 years)	-1.4	(-3.5, 0.6)
Rural residence	**13.9**	**(6.2,21.7)**
Mother's education (tertile)	**13.5**	**(11.1,15.9)**
Improved drinking water	-1.7	(-5.9, 2.5)
Improved toilet facility	-3	(-7.9, 1.9)
3rd dose of DTP vaccine	**7.7**	**(5.7, 9.6)**
Vitamin A	**35.1**	**(31.6,38.6)**

Bolded findings indicate a relationship with a $p<0{\cdot}05$. Marginal effects refer to absolute change in mean deworming coverage associated with one unit change in variable. Robust standard errors clustered by country and survey. See Methods for statistical specifications. DTP; diphtheria, tetanus, and pertussis
